# Light management by algal aggregates in living photosynthetic hydrogels

**DOI:** 10.1073/pnas.2316206121

**Published:** 2024-05-28

**Authors:** Sing Teng Chua, Alyssa Smith, Swathi Murthy, Maria Murace, Han Yang, Lukas Schertel, Michael Kühl, Pietro Cicuta, Alison G. Smith, Daniel Wangpraseurt, Silvia Vignolini

**Affiliations:** ^a^Yusuf Hamied Department of Chemistry, University of Cambridge, Cambridge CB2 1EW, United Kingdom; ^b^Marine Biology Section, Department of Biology, University of Copenhagen, Helsingør DK-3000, Denmark; ^c^School of Chemical Engineering, University of Chinese Academy of Sciences, Beijing 100040, China; ^d^Seprify AG, Marly 1723, Switzerland; ^e^Cavendish Laboratory, University of Cambridge, Cambridge CB3 0HE, United Kingdom; ^f^Department of Plant Sciences, University of Cambridge, Cambridge CB2 3EA, United Kingdom; ^g^Marine Biology Research Division, Scripps Institution of Oceanography, University of California San Diego, La Jolla, CA 92093-0205; ^h^Department of Nanoengineering, University of California San Diego, La Jolla, CA 92093-0205; ^i^Sustainable and Bio-inspired Materials, Max Planck Institute of Colloids and Interfaces, Potsdam 14476, Germany

**Keywords:** hydrogels, living materials, photosynthesis, optical modeling

## Abstract

Rapid progress in algal biotechnology has triggered a growing interest in hydrogel-encapsulated microalgal cultivation, especially for the engineering of functional photosynthetic materials and biomass production. An overlooked characteristic of gel-encapsulated cultures is the emergence of cell aggregates, which are the result of the mechanical confinement of the cells. Such aggregates have a dramatic effect on the light management of gel-encapsulated photobioreactors and hence strongly affect the photosynthetic outcome. To evaluate such an effect, we experimentally studied the optical response of hydrogels containing algal aggregates and developed optical simulations to study the resultant light intensity profiles. The simulations are validated experimentally via transmittance measurements using an integrating sphere and aggregate volume analysis with confocal microscopy. Specifically, the heterogeneous distribution of cell aggregates in a hydrogel matrix can increase light penetration while alleviating photoinhibition more effectively than in a flat biofilm. Finally, we demonstrate that light harvesting efficiency can be further enhanced with the introduction of scattering particles within the hydrogel matrix, leading to a fourfold increase in biomass growth. Our study, therefore, highlights a strategy for the design of spatially efficient photosynthetic living materials that have important implications for the engineering of future algal cultivation systems.

## Significance

Light distribution within algal cultures is one of the primary limitations to scalable and efficient biomass growth, a pertinent issue given the increasing interest in nonplanktonic growth methods, such as biofilms. Within these, cells experience uneven illumination via either overexposure on the outer surface or underexposure inside the film. We show how light distribution is altered upon cell aggregation, which naturally occurs under confinement, and enhanced through the incorporation of scatterers. Our work provides insights into how future photobioreactors could be engineered to optimize light delivery, allowing efficient cultivation of microalgae at scale. Last, our work also provides a better understanding of light propagation through gel-encapsulated biomass, a key area given the rise of research interest in engineered living materials.

The escalating demand for novel materials with biomimetic functionalities has stimulated the development of so-called biohybrid systems, which are typically composed of a soft hydrogel matrix hosting living cells that can perform various functions (1–4). Biohybrids incorporating photosynthetic organisms such as photosynthetic bacteria or microalgae have been proposed for diverse applications, ranging from chemical sensing (5–7), bioremediation (8), biotransformation (9), cell regeneration (10), bioelectronics (11, 12), hydrogen generation (13), and energy production by artificial leaves (14).

Algal-based biohybrid systems offer a highly effective platform for algal cultivation, mitigating several fundamental challenges associated with traditional photobioreactors, where the algae are planktonic, i.e., freely dispersed in liquid suspension cultures (15–17). From notable improvements in space and water requirements (18, 19) to protection against contamination (20, 21) and environmental stress (22), studies have reported that biohybrids for algal cultivation show enhancement in photosynthesis (23) and growth rates (24), as well as increased production of secondary metabolites such as pigments and lipids (25, 26) when compared to traditional methods. In addition, gel-encapsulated cultures provide distinct advantages for specific applications such as carbon capture (27), water treatment (28–30), and noninvasive metabolite harvesting (31), while preventing contamination of surrounding natural water systems and potential threats to native species (32).

However, for photosynthetic systems to function efficiently, it is crucial to achieve a homogeneous distribution of light throughout the entire material, while minimizing both overexposure and self-shading (33, 34). Nonetheless, there is a lack of studies investigating light delivery in such hybrid living systems, or the underlying reasons for the observed improvements in performance. Most studies on light management in algae cultures have focused on liquid suspension cultures, where algal cells are homogeneously dispersed either freely in an aqueous phase (35–38) or within a hydrogel matrix (39), rather than growing naturally into aggregates, which is instead what occurs when algae are encapsulated (23, 40). Thus, how aggregate formation impacts light propagation and photosynthetic efficiency in gel-immobilized algal cultures and photobioreactors remains a compelling and intriguing question that requires thorough investigation through optical modeling and experimental analysis.

In this study, we performed optical characterization of the microalgal biomass confined within a hydrogel matrix and studied how cell aggregation affects light management. More specifically, we measured the transmittance of light through agarose gel pads containing algal aggregates and compared the experimental values with predictions from Monte Carlo–based modeling of radiative transfer (41, 42). The simulated local scalar irradiance was coupled into a net photosynthetic rate (*P_net_*) model based on the Harrison model (43), providing insight into the expected *P_net_* in homogeneous algal biofilms and encapsulated algal aggregates in photobioreactor systems. We explored different incident irradiance levels and variable areal biomass densities, considering the impact of aggregate growth within the hydrogel. We also studied how cell seeding density and the scattering properties of the matrix affected light propagation, both computationally and experimentally. The results highlighted a fundamental difference in terms of light distribution between a homogenous biofilm and a gel-embedded distribution of algal aggregates, while also demonstrating a potential for further light-harvesting enhancement via modulating the scattering properties of the hosting matrix. The understanding of light propagation within hydrogel-based systems is vital for the optimization of photosynthetic performance in applications such as biophotovoltaics (12, 44) and biohydrogen production (45). Our findings also have significant implications for the optimization of light transport within immobilized algal cultures and the design of photobioreactors for microalgae cultivation and harvesting, which are now becoming increasingly important due to the growing commercial demand for sustainable food sources and additives (25, 46).

## Results

### Optical Characterization of Immobilized Biomass.

**Fig. 1.**
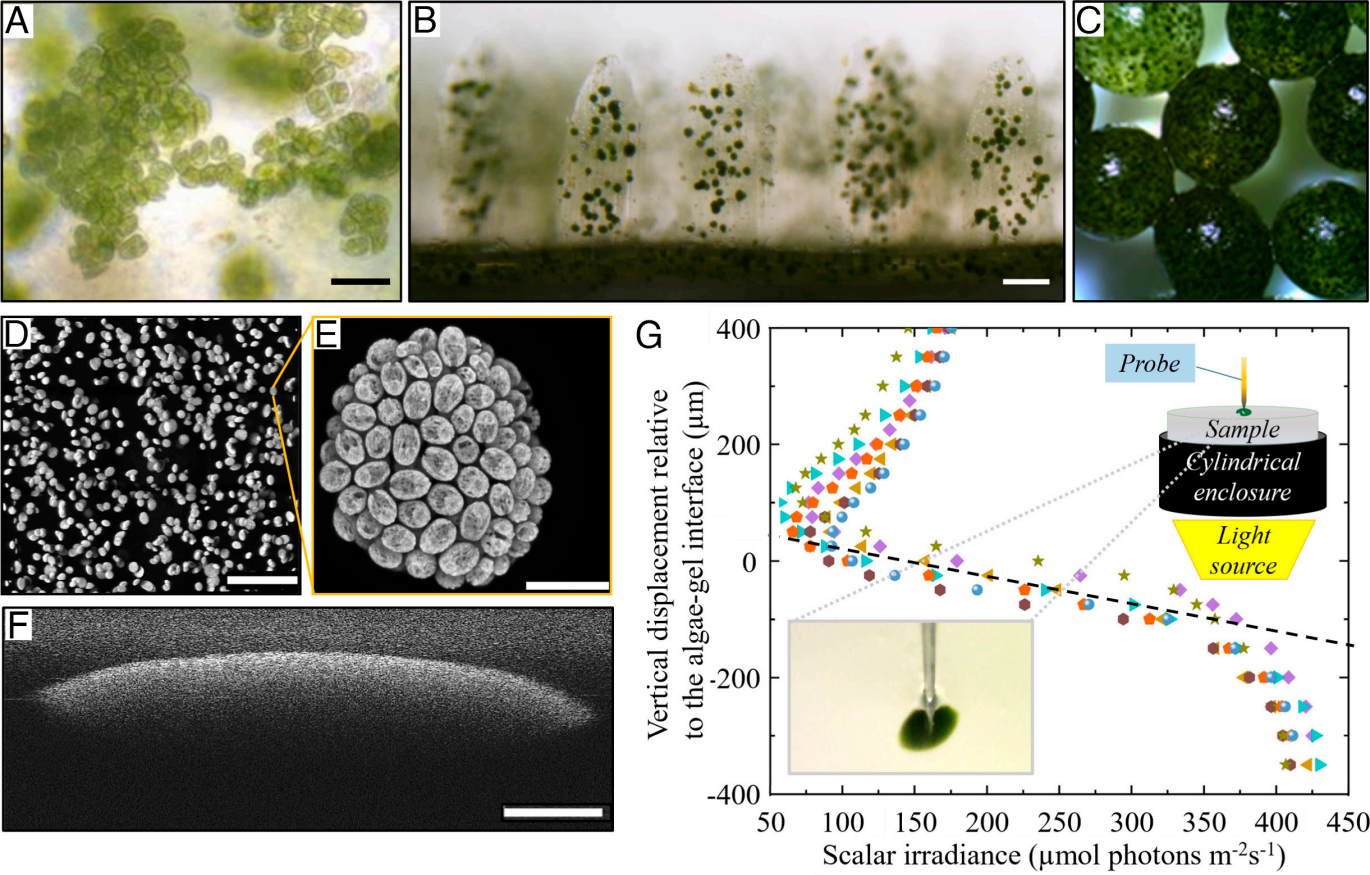
Cell aggregation resulting from growth of gel-immobilized green microalgae: (*A*) Isolated colonies of Platymonas sp. in silk-based hydrogel (Scale bar, 10 µm) adapted with permission from ref. 48 under Copyright 2023 American Chemical Society; (*B*) Aggregates of Marinichlorella kaistiae KAS603 in a 3D-printed bionic coral (Scale bar, 100 µm) reprinted from ref. 23 under Creative Commons CC-BY 4.0; (*C*) Tight clusters of *C. vulgaris* encapsulated within sodium alginate (reprinted from ref. 27 under Creative Commons CC-BY 4.0); (*D*) Confocal imaging (mean Z-stack projection) of chlorophyll autofluorescence emitted from *C. reinhardtii* aggregates after 7 d of growth within an agarose hydrogel (Scale bar, 1 mm); (*E*) Close-up maximum intensity projection of a Z-stack through a single aggregate (Scale bar, 25 µm); (*F*) Cross-sectional view of an immobilized algal aggregate imaged with an OCT system (Scale bar, 500 µm); (*G*) Microsensor profile of photon scalar irradiance (400 to 700 nm) measured across individual *C. reinhardtii* aggregates, where the origin was set to be the upper interface between the aggregate and hydrogel matrix. The *Top*-*Right* *Inset* shows the schematic illustration of the microsensor measurement setup. The *Bottom*-*Left* *Inset* illustrates a close-up photograph of the microsensor tip penetrating an isolated algal aggregate.

The encapsulation of microalgae within a hydrogel matrix inevitably leads to the formation of dense aggregates, primarily caused by cell division and the inability of the daughter cells to disperse within the physical confinement imposed by the encapsulating matrices (23, 27, 47, 48). This phenomenon is general, as the algae are physically constrained, and it can be observed in a wide variety of biohybrids composed of different types of algae and hydrogels (Fig. 1 *A*–*C*).

In this study, we evaluate the capability of light management within such biohybrids by considering model systems of the green microalga *Chlamydomonas reinhardtii* encapsulated in agarose gel. However, many of the considerations we presented can be extended to other types of biohybrids. The first step for modeling realistic experimental conditions was to evaluate the precise shape of the aggregates and their scattering capability. To achieve this, we exploited both confocal microscopy and optical coherence tomography (OCT) techniques, revealing that the aggregates developed a lenticular shape (Fig. 1 *D*–*F*). To conduct optical simulations, we used a simplified model where the gel-encapsulated culture was represented as a random arrangement of spherical algal aggregates embedded within the hydrogel matrix. This approximation was made based on the observation that the oblateness of the aggregates had minimal effects on the optical attenuation results (*SI Appendix*, Fig. S1).

To evaluate the scattering parameters, we performed OCT with 930 nm light on isolated microalgal aggregates and estimate the scattering coefficient $\mu_{s}$ and anisotropy factor g within the confined biomass (Fig. 1*F* and *SI Appendix*, Fig. S2). The empirical fitting of the backscattered intensity suggested that the values of $\mu_{s}$ and g for 930 nm within individual algal aggregates were 1,000 ± 100 cm^−1^ and 0.99, respectively. To increase the accuracy of empirical fitting, we required sufficient aggregate thickness for attenuation of backscattered intensity. As a result, we intentionally cultivated substantially larger aggregates than under standard growth conditions by controlling inoculation density for use in the OCT measurements. For instance, the aggregate featured in Fig. 1*F* had a width of 2.7 mm and a thickness of 0.45 mm. More details on how these values were extracted from the OCT data are reported in *SI Appendix*.

To assess the extent of light attenuation, the variation of spectral scalar irradiance was measured within individual aggregates, as a function of depth beneath the algae-gel interface. Considering the integrated spectral range from 400 to 700 nm, which corresponds to the photosynthetically active radiation (PAR), the value of the scalar irradiance extinction coefficient $\mu_{ext,\;PAR}$ was calculated from the slope of log-transformed light attenuation curves (Fig. 1*G*) and found to be 180 ± 20 cm^−1^ based on the following equation (49):

[1] $$\mu_{ext,\;PAR}=\frac{ln(\frac{I_{a}}{I_{b}})}{z_{a}-z_{b}}.$$

The scalar irradiance attenuation is equal to the absorption coefficient divided by the average cosine of all the incident photons at the medium interface (50). Considering that the algal biomass was highly forward scattering, assuming minimal wavelength dispersion of scattering anisotropy, the attenuation coefficient measured from scalar irradiance was close to the absorption coefficient, within the percentage uncertainty of measurement. Hence, we determined the absorption coefficient $\mu_{a}$ to be 180 ± 20 cm^−1^. Notably, this estimation aligns closely with the reported values from microalgal biofilms (51), suggesting that the extent of optical attenuation within isolated individual aggregates is comparable to that observed in dense biofilms.

### Simulation of Light Propagation through Gel-Immobilized Aggregates.

**Fig. 2.**
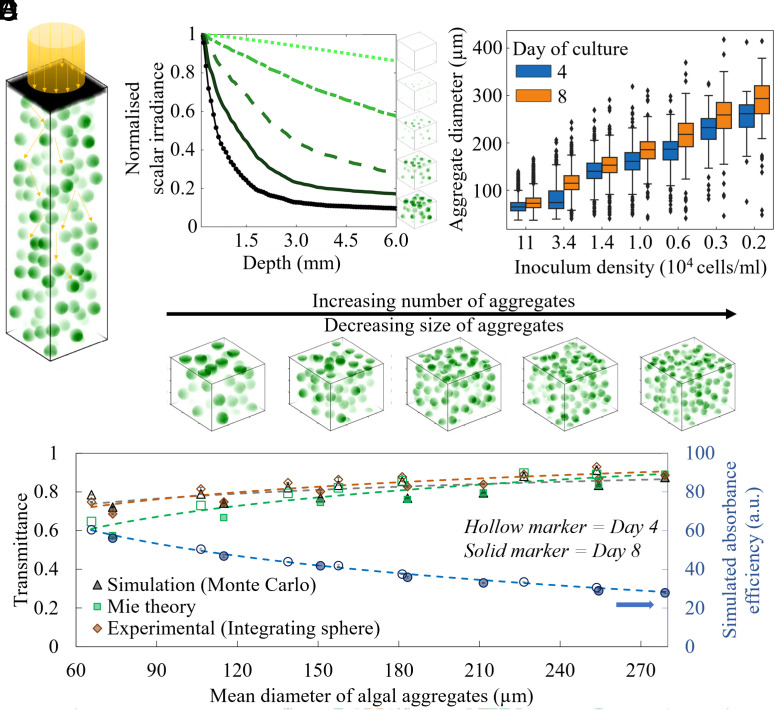
(*A*) Schematic of modeled configuration in the Monte Carlo simulation showing the downwelling beam illuminating microalgal aggregates, here represented as 10 to 100 µm wide spheres randomly distributed within the agarose hydrogel; (*B*) Simulated attenuation of scalar irradiance normalized against the incident value as a function of depth in a hydrogel matrix with *C. reinhardtii* aggregates of different sizes but constant density; (*C*) Quantification of algal aggregate size distribution with varied inoculum density using confocal microscopy on day 4 and day 8 during cultivation; (*D*) Illustrative example of constant algal density with fewer large algal aggregates and a greater number of small algal aggregates; (*E*) Comparison of total transmittance measured with an integrating sphere on hydrogel samples with different algal aggregate size distributions on day 4 (hollow marker) and on day 8 (solid marker) with the Monte Carlo simulations and the analytical calculations with Mie’s theory and Beer–Lambert’s law. The secondary axis illustrates the simulated values of the overall absorbance efficiency, marked by blue circles.

To simulate the effect of light propagation through gel-immobilized aggregates, it is important to consider that the system was not static, but was evolving continuously, with gel-encapsulated colonies developing into aggregates from individual algal cells that underwent cell growth and division. Therefore, different growth stages were simulated with different aggregate sizes under constant aggregate positions and numbers (Fig. 2 *A* and *B*). As expected, we observed a reduction in normalized scalar irradiance with the simulation depth, indicating greater light attenuation through absorption and scattering. The growth of the aggregate gave rise to greater attenuation of light with depth, due to the increased number of algae absorbing light (Fig. 2*B*). It is also important to notice that the intensity plateaued on a nonzero value, indicating that light was also scattered more efficiently and not fully absorbed.

To validate the simulation results of light transmission experimentally, we studied the effect of aggregate size and density of *C. reinhardtii* using integrating sphere measurement and quantitative analysis of aggregates through confocal imaging (Fig. 2*C*). To prepare samples with algal aggregates of varying size and density distribution, we inoculated the culture with varying cell densities. Specifically, we observed that a lower inoculum density led to the formation of sparser but larger algal aggregates, while a larger inoculum density led to smaller aggregates (Fig. 2 *C* and *D*). A mixotrophic culture with Tris-acetate-phosphate (TAP) medium was established in agarose gels such that the upper limit of aggregate size was extended as much as possible to a size that would not be reached with a Tris-minimal medium. This was done to match the model as closely as possible. Fig. 2*C* also shows that independently from the inoculation density, the size of the aggregates increased from day 4 to day 8, indicating that the cells were growing and dividing actively during this cultivation period.

Transmittance spectra of gel-encapsulated cultures for all the different inoculation conditions were measured after 4 and 8 d of growth (Fig. 2*E*). Here, we observed that light attenuation was more pronounced when aggregates were smaller and denser, while larger more dispersed aggregates gave a lower light attenuation. To evaluate the effective light absorbance for these different conditions, we performed Monte Carlo simulations: we considered numerous algal aggregates encapsulated within hydrogel culture, according to the spatial distribution and size variance of the algal aggregates obtained with confocal microscopy. As a result, we observed that the formation of sparser, but larger algal aggregates contributes to higher optical transmittance, as supported by the numerical simulation and Mie theory prediction. The numerical simulation and experimental results only deviated slightly from the theoretical prediction, which computed the effective attenuation coefficient using Mie’s theory and calculated the overall light transmission with Beer–Lambert’s law, (as the assumption of homogeneous media implied in Beer–Lambert’s law did not hold and could not account for localized shading of light within individual aggregates). Algal aggregates that were larger and sparser provided less effective light absorbance per unit biomass volume, as expressed in our simulations by the lower percentage of the incident photon energy deposited per unit voxel (Fig. 2*E*).

### Comparison of Light Transmission and Utilization within Algal Biofilm and Gel-Immobilized Algae.

In contrast to planktonic cultures, biofilms and gel-immobilized cultures of microalgae give rise to higher biomass production and facilitate harvesting (1, 52). We, therefore, compared the capacity of the total areal biomass production per volume in these two surface-associated configurations of microalgae.

**Fig. 3.**
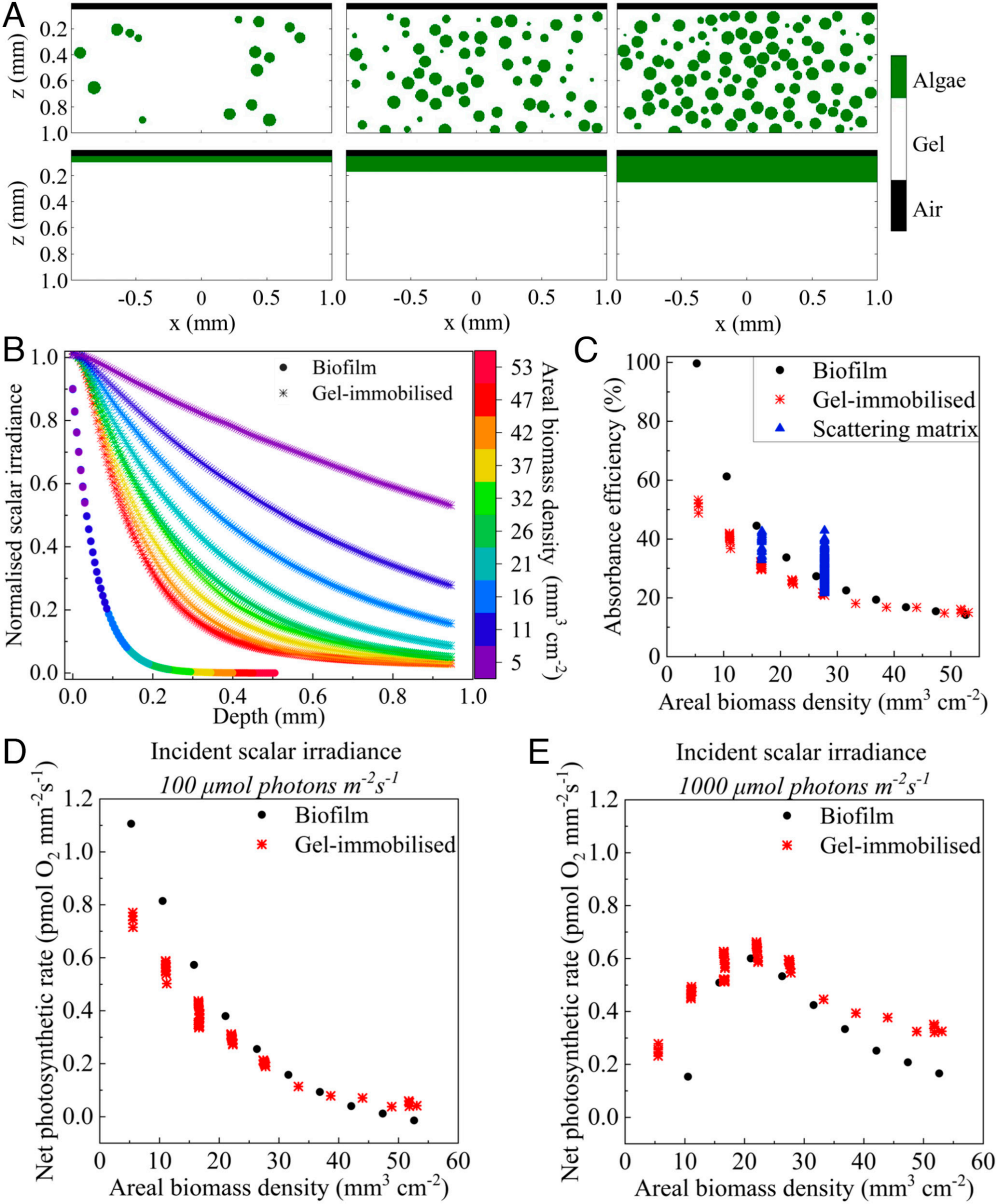
(*A*) Schematic cross-sections of gel-immobilized culture (*Top*) and biofilm (*Bottom*) with equal areal biomass density used in simulation; (*B*) 3D Monte Carlo simulation of scalar irradiance attenuation across biofilm (represented by ●) of varied thicknesses and across the hydrogel culture (represented by ∗) with a mean aggregate radius of 50 µm but different aggregate densities, corresponding to varying areal biomass densities as represented by the color scale; (*C*) calculation of the total light absorbance efficiency and the net photosynthetic rate (*P_net_*) by coupling the experimental light response curve to the simulated variation of scalar irradiance with depth in the algal aggregate containing matrix and the microalgal biofilm for different areal biomass densities under an incident scalar irradiance of (*D*) 100 and (*E*) 1,000 µmol photons m^−2^ s^−1^.

We modeled the gel-immobilized cultures and biofilm with an equal areal biomass density so that the total amount of algae and surface area of illumination were kept constant in all scenarios (Fig. 3*A*). The simulation model considered lateral light loss with photons escaping in all directions, i.e., top, bottom, and four sides of the simulation volume. The lateral dimension was chosen to be at least twice that of the simulation depth, to minimize simulation artifacts in the form of edge effects. For a better comparison across varied areal biomass densities, the illuminated area of the simulation volume was kept constant at z = 1 mm and x = y = 2 mm. However, different biofilm thicknesses were explored from z = 0.5 mm to 5 mm depth, keeping a constant area of illumination (x = y = 2 mm) and the same areal density of algal biomass in gels compared to biofilms. A higher areal biomass density corresponded to a thicker biofilm and a denser aggregate distribution in hydrogel immobilization, and vice versa.

Owing to the dense distribution of absorbers (algae) in a biofilm, the normalized scalar irradiance approached zero at a depth of 200 µm (Fig. 3*B*), consistent with previous reports, where biofilm growth was typically limited to a thickness of 200 µm to 300 µm (51, 53, 54). In contrast, the gel-encapsulated system exhibited much less light attenuation (Fig. 3*B*). Given a high enough areal biomass density, the gel-immobilized algal aggregates eventually exhibited light attenuation similar to the one observed in biofilm via self-shading among aggregates. At very high biomass densities, the aggregates tended to overlap and coalesce, forming a network of interconnected biomass, that could be observed in practical experiments (*SI Appendix*, Fig. S3).

The understanding of light propagation and absorbance alone was, however, insufficient to characterize biomass production, as the photosynthetic rate is not linear with the amount of light absorbed. To correlate the effective scalar irradiance to microalgae photosynthetic activity across the simulation volume depth, we coupled the optical simulation outcome to an experimentally determined light response curve fitted to an empirical model (*SI Appendix*, Fig. S4), taking the effect of photoinhibition into account. Specifically, we used the Harrison model (43) for estimating the net photosynthetic rate *P_net_*, based on a known value of local scalar irradiance *I*:

[2] $$P_{net}=P_{sat}\text{exp}\left(\frac{-\beta I}{P_{sat}}\right)\left[1-\exp\left(\frac{-\alpha I}{P_{sat}}\right)\right]-R_{d},$$

where $P_{sat}$ indicates the maximum *P_net_*, while α and β represent the light-limited initial slope and photoinhibition constant, respectively. The rate of dark respiration is represented by *R_d_*.

*P_net_* was computed for all configurations using a range of incident irradiance levels from 100 to 2,000 (natural sunlight) µmol photons m^−2^ s^−1^ (*SI Appendix*, Fig. S4). Experimental determination of *P_net_* was done via oxygen microsensor measurements. Comparisons were made between the biofilm and aggregate system by integrating *P_net_* across all depths, before normalizing it by biomass density.

At low incident irradiance (100 µmol photon m^−2^ s^−1^), the integrated *P_net_* followed the trend of light absorbance efficiency in general (Fig. 3*C*), as it fell in the light-limiting regime, with higher biomass density resulting in lower *P_net_* due to greater light attenuation (Fig. 3*D*). The biofilm generated higher *P_net_* compared to the gel-immobilized system up to the thickness threshold where the light was attenuated completely in the biofilm. Beyond this threshold, around 35 mm^3^ cm^−2^ in areal biomass density, the gel-immobilized configuration exhibited slightly higher *P_net_*, as the bottom of the biofilm was heavily shaded, while the gel-immobilized system still had some light reaching the aggregates at the bottom, allowing for moderate photosynthetic activity.

Under high incident irradiance (1,000 µmol photon m^−2^ s^−1^), photoinhibition became significant, as predicted by the Harrison model (*SI Appendix*, Fig. S5). Given high absorbance efficiency and less shielding effect at low areal biomass density, more cells were photoinhibited, giving rise to lower *P_net_*. On the other hand, with the increase of areal biomass density, the extent of self-shading increased, lowering the degree of photoinhibition, until the point of light saturation for maximal photosynthetic capacity. With further self-shading, part of the shielded culture became light-limited (Fig. 3*E*). While the top layer of the biomass may be photoinhibited, the shaded biomass would be exposed to the optimal light level. Overall, the gel-immobilized system exhibited higher *P_net_*, indicating a lower degree of photoinhibition, as compared to the biofilm system.

### Performance Optimization via the Scattering Properties of the Hydrogel Matrix.

**Fig. 4.**
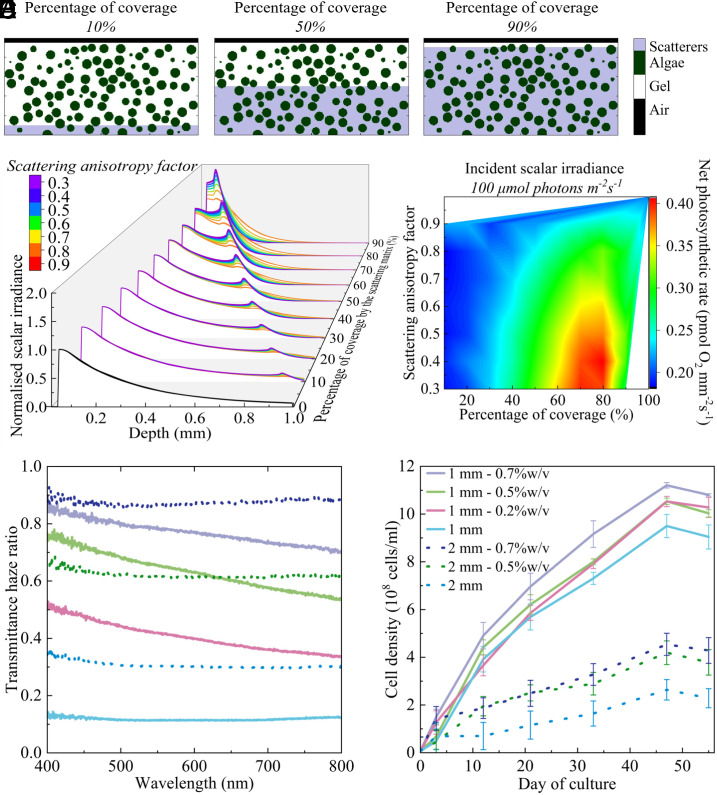
(*A*) Schematic cross-sections of varied scattering matrix coverage in percentage, with the scatterers being concentrated near the bottom boundary; (*B*) 3D Monte Carlo simulation of light attenuation across the hydrogel culture with a mean aggregate radius of 50 µm and an areal biomass density of 27 mm^3^ cm^−2^, but varying scattering matrix coverage and scattering anisotropy represented by the color scale; Calculation of the net photosynthetic rate (*P_net_*) by coupling the experimental light response curve to the simulated variation of normalized scalar irradiance among algal aggregates within different scattering matrix configurations under an incident scalar irradiance of (*C*) 100 µmol photons m^−2^ s^−1^; (*D*) The effect of CMP doping on the transmittance haze across a 1 mm- (solid line) and 2 mm- (dotted line) thick agarose hydrogel with different weight percentage of embedded CMP; (*E*) The cell growth curve of *C. vulgaris* in 1 mm- (solid line) and 2 mm- (dotted line) thick hydrogels, color matched to the CMP doping level it represents.

To increase the absorbance efficiency, and therefore increase the probability of interaction between photons and algal aggregates, we explored the effect of incorporating scattering particles within the hydrogel matrix. While a heterogeneous biomass distribution would enable the transmission of some photons among cell aggregates without interaction with the biomass, a scattering matrix with a wide angular range of scattering directions could redirect these unabsorbed photons to interact with the algal aggregates, effectively improving the probability of light absorption. Experimentally, such enhancement of the scattering efficiency of the matrix can be achieved by incorporating scattering particles into the hydrogel matrix. Fig. 3*C* shows that increasing the scattering efficiency in the surrounding matrix increased the effective absorbance per aggregate beyond that of the biofilm at high areal biomass density. A key parameter to consider when adding scattering particles in the matrix is their filling fraction and distribution. The simplest approach is to use a uniform dispersion of scatterers throughout the hydrogel matrix. The resulting scattering coefficient of the matrix scales with the overall concentration of scatterers per unit volume. Alternatively, further optimization could be achieved by concentrating scatterers at different depths of the hydrogel matrix, as illustrated in Fig. 4*A*.

Our simulations showed that addition of scattering particles to the hydrogel matrix with different anisotropy factors and percentage of coverage affected the light distribution within the matrix (Fig. 4*B*). In contrast to a smooth exponential decay of normalized scalar irradiance in a material without a scattering matrix (represented by the black line), the interface between a normal hydrogel matrix and a scattering matrix created a rippled intensity profile, as a result of enhanced backscattering. The degree of this enhancement was influenced by the anisotropy factor of the scattering particles, with a lower anisotropy factor resulting in more pronounced backscattering effects. We note that following the peak of enhanced scalar irradiance at the medium-gel interface, light availability in deeper parts of the hydrogel was diminished, as the scattered photons were either absorbed by the algae or escaped from the upper interface. The associated shifts in the profile of normalized scalar irradiance affected the overall absorbance efficiency and hence the integrated *P_net_* of the entire biomass volume, depending on the incident irradiance (Fig. 4*C* and *SI Appendix*, Fig. S6). In the hydrogel-immobilized system, mutual shading among the aggregated cells resulted in the concentration of photons in the upper region, as light is attenuated with depth. Hence, under low-incidence irradiance, increasing the availability and absorbance of photons in the upper region significantly enhanced the overall photosynthetic efficiency, particularly with a higher percentage of scatterers with a lower anisotropy factor (Fig. 4*C*).

The distribution of the scatterers is also important. Excessive backscattering especially in the upper region of the hydrogel caused photoinhibition and reduced photon transfer to the lower regions when the scatterer coverage exceeded 70%. This was demonstrated in the case of 90% scatterer coverage, where the maximum scalar irradiance reached up to twice the incident irradiance (Fig. 4*B*). Moreover, since under high incident irradiance, the algae in the upper region of the hydrogel were subjected to photoinhibition without any scattering presence, scattering enhancement in the upper area of the hydrogel would lower the overall net photosynthetic efficiency. In contrast, the scattering enhancement was optimal in the light-limiting regime, namely the middle zone (40 to 50% scatterer coverage from the bottom of the gel) after some degree of light attenuation from mutual shading (*SI Appendix*, Fig. S6*A*). With an even higher irradiance (1,000 µmol photon m^−2^ s^−1^), the optimal zone for scattering matrix coverage reduced to the lower region of 20 to 40% scatterer coverage from the bottom of the gel, as the light-limiting regime was shifted further downward (*SI Appendix*, Fig. S6*B*). Under high irradiance, if the scattering interface was in the upper region with 80% coverage in depth, the *P_net_* decreased with a lower anisotropy factor as photoinhibition was aggravated with higher photon interaction in the light-saturated region (*SI Appendix*, Fig. S6 *C* and *D*).

As a proof-of-concept experiment, agarose hydrogels were embedded uniformly with cellulose microparticles (CMP) (55) to increase light scattering within the hydrogel matrix. CMPs were chosen as scattering particles for their optimized scattering abilities and their biocompatible characteristic. As shown in *SI Appendix*, Fig. S7, the addition of CMP resulted in increased opacity of the hydrogel, owing to a higher ratio of the haze transmittance to the total transmittance with higher proportions of CMP (Fig. 4*D* and *SI Appendix*, Fig. S8). CMP doping introduced significant light scattering within the hydrogel matrix. Within the 1 mm-thick hydrogels, 0.7%w/v CMP doping caused 75 to 85% of the transmitted light to be scattered, as compared to ~15% in standard hydrogels without CMP (0%w/v). When comparing the spectra of the 2 mm hydrogels, 0.7%w/v CMP doping scattered approximately 90% of the transmitted light. This demonstrates that the addition of CMP altered the light profile throughout the hydrogels, thereby influencing the growth of immobilized microalgae (Fig. 4*E*). The versatility of this approach was evident across microalgal species. A similar result to that for *C. reinhardtii* was observed (*SI Appendix*, Fig. S9) using another green alga, *Chlorella vulgaris,* which is widely used for large-scale cultivation in commercial applications.

Within a 1 mm-thick agarose pad containing 0.7%w/v CMP, higher biomass was observed throughout the entire growth curve, showing an approximate 50% increase in cell numbers after 10 d, in comparison to pads without CMP. Additionally, there was cumulatively improved growth in the agarose with 0.7%w/v CMP, rather than an initial increase, which was then maintained. In 2 mm-thick agarose pads, both 0.5%w/v and 0.7%w/v CMP doping resulted in a higher degree of growth enhancement, with approximately 100% increase in cell numbers after 10 d (Fig. 4*E*). Comparing the cell density alone, the 1 mm-thick hydrogels were much more productive than the 2 mm-thick hydrogels, with or without scattering, most likely as a result of gas and solute exchange being limited by increasing hydrogel thickness.

## Discussion

By comparing the light management capabilities of biofilms and gel-immobilized cultures, we conclude that gel-immobilized algal cultures have the potential to reach a higher areal biomass density compared to flat, homogeneous algal biofilms. Our results suggest that the formation of cell aggregates upon hydrogel immobilization is crucial, as it reduces the probability of photon-cell interaction, effectively lowering the scattering and absorption coefficient (56–58). As a result, more photons were able to penetrate and reach greater depths in the gel-immobilized algal culture (Fig. 3*B*). Such an increase in light penetration depth alleviated the self-shading of the algal biomass. Such self-shading is inevitable in dense microalgal biofilms, limiting their thickness to 300 µm or less (51, 53, 54), corresponding to an aerial biomass density of ~30 mm^3^ cm^−2^.

Additionally, gel-immobilized systems were able to achieve significantly higher *P_net_* than biofilms at higher biomass densities, particularly when higher incident irradiance was required to counteract self-shading within the growing biomass (Fig. 3 *D* and *E*). While photoinhibition could be minimized with a lower incident irradiance, the predominance of a light-limiting regime would lower the overall photosynthetic efficiency, especially at high biomass density with significant self-shading (Fig. 3*D*). Hence, as the biomass density increased, moderate to high levels of illumination could reach the shaded cells in the lower region of the gel-immobilized algal culture. This conclusion is supported by the decrease in overall *P_net_* with biomass density and the enhancement of *P_net_* with incident scalar irradiance beyond an aerial biomass density of 20 mm^3^ cm^−2^ (Fig. 3*D*). In comparison to a homogeneous biofilm, a gel-immobilized culture achieved higher *P_net_*_,_ with an aerial biomass density exceeding 30 mm^3^ cm^−2^. A heterogeneous biomass distribution from cell aggregation reduced the proportion of photoinhibited cells among the top layers as not all cells located in the upper layers were exposed to the same irradiance level. Furthermore, self-shading within individual aggregates protected some of the cells against excessive irradiance. In contrast, the algal biomass in biofilms was uniformly exposed and hence equally photoinhibited in the top layer. With further self-shading, part of the shielded culture became light-limited (Fig. 3*E*). Meanwhile, the shaded biomass beneath the top layer received an optimal light level. In the case of gel immobilization, the percentage of photoinhibited biomass was significantly lower compared to the biofilm, owing to its heterogeneous distribution of cells. Such optimization required finding an optimal trade-off between alleviating the light shading in the lower region with intense illumination irradiance while incurring a smaller degree of photoinhibition in the upper region of biomass.

Finally, a gel-immobilized system was able to deliver light more efficiently than a biofilm even when a low incident irradiance is desired, given an optimal scattering matrix. We found that modifying the scattering properties of the hydrogel matrix could enhance the overall photosynthetic performance, both in our simulations (Fig. 4*C*) and experiments (Fig. 4*E*). Algal growth in gel-immobilized systems with added scattering particles was promoted through increasing the amount of light available for photosynthesis. This is especially prominent given that these cultures were grown under low photon irradiance (~40 µmol photons m^−2^ s^−1^). In industrial settings, direct and intense light sources cannot always be guaranteed, and factors like shading or variable sunlight can dilute the light reaching the samples. With the proof of concept shown here, it is now possible to develop a light-sensitive material with dynamic scattering properties upon light exposure of varying intensity, optimizing the light distribution with a better trade-off between the proportion of photolimited and photoinhibited cells within the algal cell population.

However, it is important to consider that light management is not the sole factor to consider in a photobioreactor. The availability of gases and nutrients in a hydrogel system depends on the diffusion of molecules within and between cell aggregates. Diffusion-limited growth became evident from our observation of a lower cell density within a thicker bulk hydrogel (Fig. 4*E*). Potential strategies have been studied to address diffusion limitations in hydrogels, such as 3D bioprinting to increase the surface area-to-volume ratio (23, 59) or cocultivation of algae with symbiotic bacteria to enhance gas and nutrient exchange (39). We also note that our study has used a simplified assumption to capture the main optical properties of a homogeneous biofilm. In reality, biofilms growing on a substrate can exhibit more complex morphology both in terms of uneven surface morphology and bulk porosity (51, 60). Notably, there are multiple factors in play concerning the photosynthetic performance of any system. This paper considers the efficiency of light harvesting and utilization for photosynthesis primarily from the perspective of the spatial distribution of algal biomass. With the advancement of cell-matrix composites using encapsulation and immobilization techniques, it is important to understand how these processes implicate optical performance, and our work serves to address the impact of cell aggregation.

In conclusion, we showcase the advantages of cell aggregation in gel-encapsulated colonies of *C. reinhardtii* compared to biofilm growth, when it comes to light management. This aggregation led to improved light transmission and utilization, particularly under optimal incident irradiance. As biomass density increased and self-shading became more prominent, the aggregated system achieved a better balance between photolimited and photoinhibited regimes when exposed to higher incident irradiance. Furthermore, the addition of scattering particles enhanced light harvesting efficiency, resulting in increased growth rates of *C. vulgaris* under low incident irradiance. By highlighting the collective improvement in light allocation throughout the hydrogel culture, our findings offer insights for optimizing the design and light use efficiency of photobioreactors and microalgae-based photosynthetic living materials.

## Materials and Methods

### Cell Culture of *C. reinhardtii* and *C. vulgaris*.

The green alga *C. reinhardtii* (wild type strain 137c) were grown mixotrophically in carbon-supplemented TAP medium (61) (Tris base: 48.4 mg L^−1^; Beijernick salts (NH_4_Cl: 375 mg L^−1^, MgSO_4_·7H_2_O: 100 mg L^−1^, and CaCl_2_·2H_2_O: 50 mg L^−1^); phosphate solution (K_2_HPO_4_, KH_2_PO_4_); Kropat’s trace elements; CoCl_2_: 1 mg L^−1^; glacial acetic acid: 0.1 vol%). Liquid cultures of *C. reinhardtii* were grown in an orbital incubator (Infors HT Multitron Pro.) at 25 °C with shaking at 100 rpm under a diurnal cycle of 12h light (100 μmol photons m^−2^ s^−1^) and 12 h dark. *C. vulgaris*, was cultured in Jaworski’s medium (JM) (62), under these conditions: 16 h at 25 °C under ~40 µmol photons m^−2^ s^−1^ and 8 h dark, at 20 °C in a Panasonic MLR-352-PE growth chamber, unshaken.

### Gel Immobilization of Microalgae.

For cell immobilization, 1% w/v agarose (Sigma-Aldrich A9045) with a low gelling temperature (26 to 30 °C) was adopted as the hydrogel matrix in this work. Exponential growth phase cells were taken from suspension cultures, grown under the same conditions, for inoculation into the hydrogels. All work was performed in a flow bench (Air Science Purair Flow-24) to maintain sterile cultures. Hydrogel cultures of *C. reinhardtii* were prepared with 5% v/v of microalgal cell suspension mixed with agarose solution at varied inoculum densities ranging between 0.1 and 1 million cells per mL prior to hydrogel solidification at 30 °C. A 400 µL aliquot of the mixture was allowed to set into a disc that fully covered the observation area (20 mm in diameter) in a 35 mm glass-bottomed µ-dish (Ibidi GmbH, Gräfelfing, Germany). *C. vulgaris* were embedded in an agarose matrix, with an inoculum density of 7 x 10^6^ cells mL^−1^. Different thicknesses (1 mm, 2 mm) of agarose (1% w/v) were fabricated in 35 mm glass-bottomed µ-dishes, allowing for in situ optical characterization while maintaining a sterile environment within the petri dish. Within the petri dish, the hydrogels were submerged under 2 mL of liquid JM, that was replenished approximately weekly.

### CMP.

To enhance scattering, CMPs (1% w/v) suspended in JM were used, added in varying amounts on a w/v % basis to create agarose with different densities of CMP (0% w/v, 0.2% w/v, 0.5% w/v, 0.7% w/v). Two different solutions were prepared to allow for different final concentrations of CMP in the agarose. A: standard JM and B: the JM with 1% CMP w/v mixture. These solutions were combined in appropriate volumes, e.g., 80% A and 20% B for a 0.2% w/v CMP hydrogel, and this was then used as the precursor mix in which the agarose was dissolved. The precursor plus agarose mixture was then autoclaved to sterilize and initiate cross-linking. The CMPs were isolated from microcrystalline cellulose (SERVA Electrophoresis), with dimensions of approximately 520 nm by 2700 nm. All concentrations of CMP were used for the 1 mm thick hydrogels, while only 0%w/v, 0.5%w/v, and 0.7%w/v were used with 2 mm thick gels. Gas diffusion was increasingly problematic as the thickness of the hydrogel increased, causing air bubbles to form within the matrix as the biomass grew. We additionally ruled out the possibility of the microalgae utilizing CMP as a carbon source, the results of which can be seen in *SI Appendix*, Table S1. To determine this we grew *C. reinhardtii* cells with Trismin and TAP, with and without CMP, in the dark for 4 d and monitored the growth. As can be seen in the table there is no significant difference in growth between the presence and absence of CMP, and therefore the CMP cannot be utilized as a carbon source.

### Optical Density Measurements.

Optical density (OD) measurements were recorded using an integrating sphere (Labsphere) connected to a spectrometer (Avantes AvaSpec-ULS-RS-TEC) and light source (Thorlabs SLS201L/M), coupled via two 1 mm core fibers (FC-1000-2-SR, Avantes) to the sphere from the light source, and from the sphere to the spectrometer. Normalized transmission measurements were taken as a means to record OD, as there was negligible reflection. All measurements were taken in a dark room. The background signal was acquired with an agarose hydrogel, without algae and the corresponding amount of CMP, also covered in 2 mL of JM, without illumination. The reference signal was taken with the same configuration, however, with the illumination light on. Before measurements, the light source was left on for an hour to allow for it to stabilize. A measurement of the haze of the hydrogels was taken to quantify the scattering. To measure this, transmission spectra were taken of the hydrogels with CMP added but no *C. vulgaris*. A second measurement in the same configuration was taken but with a port removed from the integrating sphere to allow for any ballistic transmission to pass out of the sphere and not be recorded as part of the spectrum. The difference between these two spectra indicates the amount of light being scattered.

To quantify the biomass growth of immobilized cultures, a standard curve between the OD and cell numbers was established. To take OD measurements, the hydrogels were referenced to agarose hydrogels without algae, with the corresponding proportion of scattering particles, also covered in 2 mL of JM. The normalized transmission was measured and given there was negligible reflection, the normalized absorption was calculated by subtracting the transmission from 1 (e.g., 100% transmission). To extract the biomass, the hydrogels were heated to 65 °C for 5 min (Grant Bio PHMT-PSC24N Thermo-Shaker), and a further 1 mL of JM was added to prevent agarose cross-linking upon cooling. This suspension was then vortexed (Cleaver Scientific) for 10 min to break up cell aggregates and cell numbers were counted using a Neubauer Improved Hemocytometer Counting Chamber.

### Cryogenic Scanning Electron Microscopy (CryoSEM).

CryoSEM images of hydrogels were taken of the 1 mm hydrogels with either no CMP added, or 70% CMP. The CMP scattering centers appeared sheet-like among the agarose polymer network. A scanning electron microscope (FEI Verios 460) equipped with a cryotransfer system (Quorum PP3010T) was used at 2.0 kV. Samples were prepared by quenching in liquid ethane, sublimated at −90 °C (2 min), and finally sputter coated with platinum (10 nm; Quorum Technologies Q150T ES).

### Confocal Laser Scanning Microscopy.

The size and organization of cells in the algal aggregates were imaged with a confocal laser scanning microscope (Leica TCS SP5; inverted DMI 6000 CS microscope base). Using a HeNe laser for excitation at 633 nm, the detection range was set to be 660 to 710 nm, thus capturing the chlorophyll autofluorescence from *C. reinhardtii* centered at 680 nm. Low magnification imaging with a 10× objective (HC PL Fluotar, NA 0.3, Leica, Germany) was used to characterize algal colony size throughout the agarose hydrogel matrix volume by performing a z-stack across varied depth levels from the glass bottom interface to the top of the hydrogel surface. For each sample, z-stack scanning was performed with tile stitching across a lateral area of 4 mm by 4 mm. Individual algal colonies within the immobilization matrix were also imaged using a 40× oil immersion objective (HCX PL APO CS, NA 1.25, Leica, Germany).

### Optical Characterization of Algal Aggregates.

We imaged individual algal aggregates confined within an agarose gel matrix using a commercially available OCT system (Ganymede II, Thorlabs GmbH, Dachau, Germany) equipped with a broadband low coherent superluminescent diode emitting a light beam centered at 930 nm, and an objective lens with an effective focal length of 18 mm and a working distance of 7.5 mm (LSM02-BB; Thorlabs GmbH, Dachau, Germany). The experimental details followed that of a previous study (63). We evaluated $\mu_{s}$ and g within algal aggregates by measuring and fitting the reflectance signal R to an exponential decay equation (64, 65).

[3] $$R\left(z\right)=\rho e^{-\mu z},$$

where ρ refers to reflectivity (unitless) at the aggregate interface, while μ represents the attenuation of light (reciprocal length).

The experimental setup for microscale light measurements is illustrated in Fig. 1*G*. Isolated colonies of *C. reinhardtii* with very low inoculum density were grown in 1%w/v agarose gel enclosed within a 35 mm glass-bottomed µ-dish. The observation dish was illuminated from below by a fiber-optic halogen lamp (KL-2500, Schott GmbH, Germany) with controlled intensity. Approaching from the top, a fiber-optic scalar irradiance microprobe with a spherical tip diameter of 40 µm (66) was used to measure depth profiles of spectral scalar irradiance in vertical steps of 50 µm at specific positions across immobilized algal aggregates (66–68). The position of the probe tip was controlled precisely using a motorized micromanipulator system controlled by custom-built software (69).

Total transmittance spectra of gel-immobilized aggregates were measured using an integrating sphere (Labsphere inc., USA). The illumination port of the integrating sphere was coupled to a stabilized Tungsten–Halogen light source (SLS201L/M, Thorlabs, USA) via a 1-mm optical fiber, giving rise to a circular illuminating beam diameter of approximately 5 mm. Similarly, a 1-mm optical fiber was used to couple the detector port of the integrating sphere to a fiber-optic spectrometer (AvaSpec-HS2048, Avantes, USA). The transmitted light intensity was normalized with respect to an agarose gel pad without algae. The spectral measurements were performed by collecting light over 1 s and averaging over three measurement cycles for each spectrum with background subtraction. All measurements were conducted as triplicates.

### Oxygen Microsensor Measurements.

We measured the O_2_ concentration profiles across gel-immobilized *C. reinhardtii* aggregates with a Clark-type O_2_ microsensor (tip size of 25 μm, 90% response time of <0.5 s, and a stirring sensitivity of ∼1%; OX-25, Unisense A/S, Aarhus, Denmark), as described previously (70). Linear calibration of the sensor readings was performed in an air-saturated and anoxic nutrient medium used for algal cultivation. The percentage of air saturation was converted to absolute oxygen concentration (µmol O_2_ L^−1^) using tabulated values of O_2_ solubility in water as a function of temperature and salinity (Ramsing and Gundersen, Unisense, Denmark; www.unisense.com) depending on the experimental temperature and salinity. All O_2_ microsensor measurements were performed in the same spatial configuration as the scalar irradiance microsensor measurements, with precise positional control using the manipulator using a smaller vertical step interval of 25 μm. Every time the illumination state was changed, we waited for 5 to 10 min to attain a new steady-state condition of the oxygen profile. The flux of oxygen production and consumption J (nmol cm^−2^ s^−1^) were determined from the slopes of oxygen profile using Fick’s first law of diffusion:

[4] $$J\left(z\right)=-D\frac{\mathrm{dC}}{\mathrm{dz}},$$

where D is the effective diffusion coefficient (cm^2^ s^−1^), while C indicates the oxygen concentration at specific positions (nmol cm^−3^). The sum of the flux out of the isolated aggregate was considered as a measure of the net oxygen production and hence the net photosynthetic rate *P_net_*. The microalgal aggregates used in these measurements were cultured in agarose gel containing Tris-minimal (TAP medium without carbon source) to ensure that the algae were solely photoautotrophic. To achieve the aggregates of sufficient size for the probing of oxygen gradient, seeded *C. reinhardtii* was cultured in an incubator under a diurnal cycle of 12 h light (100 μmol photons m^−2^ s^−1^) and 12 h dark at 25 °C for 5 d prior to measurements.

### Three-Dimensional Voxel-Based Monte Carlo Simulation of Photon Transport.

We adapted the three-dimensional Monte Carlo simulation program *mcxyz* (41) for our modeling of radiative transfer. The simulation began by “launching” photons sequentially with an equal starting weight, which then propagated with a step size *s* that was determined stochastically. By tracking the photon weight accumulated at individual voxels depending on the optical properties, the light distribution could be simulated accordingly. The numerical approach of Monte Carlo required an accurate input of wavelength-specific optical parameters including refractive index n, scattering coefficient $\mu_{s}$, absorption coefficient $\mu_{a}$_,_ and the anisotropy factor g of both the hydrogel matrix and encapsulated microalgae. The optical parameters determined experimentally were fed into the *mcxyz* program for the simulation of light propagation within different configurations. It allowed the creation of a 3D Cartesian grid of voxels with heterogeneous distribution of media. The input file was first created via MATLAB to specify the simulation volume in terms of the number of bins and bin size, and the spatial arrangement of different medium types. The output data were then generated as a 3D array holding the spatial distribution of normalized scalar irradiance. The absorbance per unit volume per incident light energy was calculated as the product of normalized scalar irradiance and $\mu_{a}$. All simulation outcomes were compiled from an average of at least five separate runs, to account for the uncertainty in the random distribution of algal aggregates within the simulated volume.

## Supplementary Material

Appendix 01 (PDF)

## Data Availability

The data that support the findings of this study are openly available in Apollo - University of Cambridge Repository at https://doi.org/10.17863/CAM.108071 (71). All other study data are included in the article and/or supporting information.
