# Supplementary material for: Light management by algal aggregates in living photosynthetic hydrogels
Source: Proc Natl Acad Sci U S A. 2024 May 28;121(23):e2316206121. doi: 10.1073/pnas.2316206121 (PMC11161743; doi:10.1073/pnas.2316206121)
Supplement: Supplementary file 1 — Appendix 01 (PDF) [file pnas.2316206121.sapp.pdf]

Supplementary Information for

Light management by algal aggregates in living photosynthetic hydrogels.

Sing Teng Chua[1*], Alyssa Smith[1*], Swathi Murthy[2], Maria Murace[1], Han Yang[3], Lukas Schertel[4], Michael Kühl[2], Pietro Cicuta[5], Alison G. Smith[6], Daniel Wangpraseurt[7,8], Silvia Vignolini[1,9]

[1]Yusuf Hamied Department of Chemistry, University of Cambridge, Cambridge CB2 1EW, United Kingdom
[2]Marine Biology Section, Department of Biology, University of Copenhagen, Strandpromenaden 5, DK-3000 Helsingør, Denmark
[3]School of Chemical Engineering, University of Chinese Academy of Sciences, 100040 Beijing, Beijing, China
[4]Seprify AG, Route de l'Ancienne Papeterie 180, 1723 Marly, Switzerland
[5]Cavendish Laboratory, University of Cambridge, CB3 0HE Cambridge, United Kingdom
[6]Department of Plant Sciences, University of Cambridge, Cambridge CB2 3EA, United Kingdom
[7]Marine Biology Research Division, Scripps Institution of Oceanography, University of California San Diego, La Jolla, CA 92093-0205, USA
[8]Department of Nanoengineering, University of California San Diego, La Jolla, CA 92093-0205, USA
[9]Max Planck Institute of Colloids and Interfaces, 14476 Potsdam, Germany

Correspondence: Silvia Vignolini
Co-correspondence: Daniel Wangpraseurt, Sing Teng Chua, Alyssa Smith

Email: sv319@cam.ac.uk
dwangpraseurt@ucsd.edu

This PDF file includes:

Figures S1 to S9
Table S1

Extraction of optical parameters using optical coherence tomography (OCT)

Inherent optical properties for the gel immobilised algal aggregates, i.e., the scattering coefficient μ_s [cm^{-1}] and the anisotropy of scattering *g*, were obtained using theoretical models of light propagation based on the inverse Monte Carlo method (1). A more detailed description of extraction of optical properties from OCT scans can be found elsewhere (2, 3).

Briefly, OCT B-scans were acquired with a resolution of 581×1024 pixels, over a fixed depth of 2.8 mm, and variable distance in the X-plane. The setup was optimised to yield the highest signal at a fixed distance of 0.4 mm from the top of the scan. Before measurements, the OCT reflectivity (*R*) was calibrated (**SI Figure 2A**) using homemade reflectance standards with an immersion oil-glass, a water-glass, and an air-glass interface. *R* values from the standards were determined using Fresnel's equation:

$$\mathrm{R} = \left(\frac{\mathrm{n_1} - \mathrm{n_2}}{\mathrm{n_1} + \mathrm{n_2}}\right)^2 \quad (1)$$

using the refractive index (*n*) for air (1), water (1.33), immersion oil (1.46), and quartz glass (1.52). The OCT signal (in decibel, dB), from the samples, was then converted to the depth-dependent *R* via a linear fit of $\log_{10}(R)$ versus OCT intensity values (see reference (3) for details).

The focus function of the objective lens was calibrated by measuring the OCT signal fall off, in steps of 0.1 mm, from either side of the focal plane (z = 0.4 mm) to z = 0 mm and 0.8 mm, respectively. The signal loss from the focal plane follows an exponential decay function. The determined *R* values from the sample scans were corrected by dividing with the exponential fit. The corrected *R* values were then plotted against sample depth (z, distance from focal volume) and fitted to the exponential decay function (**SI Figure 2B**):

$$\mathrm{R(z)} = \rho \times \mathrm{e}^{-\mu.\mathrm{z}} \quad (2)$$

where ρ (dimensionless) is the light intensity and μ is the signal attenuation (cm^{-1}) from the focal volume. The fit was considered satisfactory if $R^2 > 0.5$.

Using the grid method (4), values of ρ and μ were mapped to *g* and μ_s based on the theory described in previous studies (3, 5). It was assumed that the sample absorption at 930 nm was negligible and that the absorption was dominated by water (μ_a ~ 0.43 cm^{-1}).

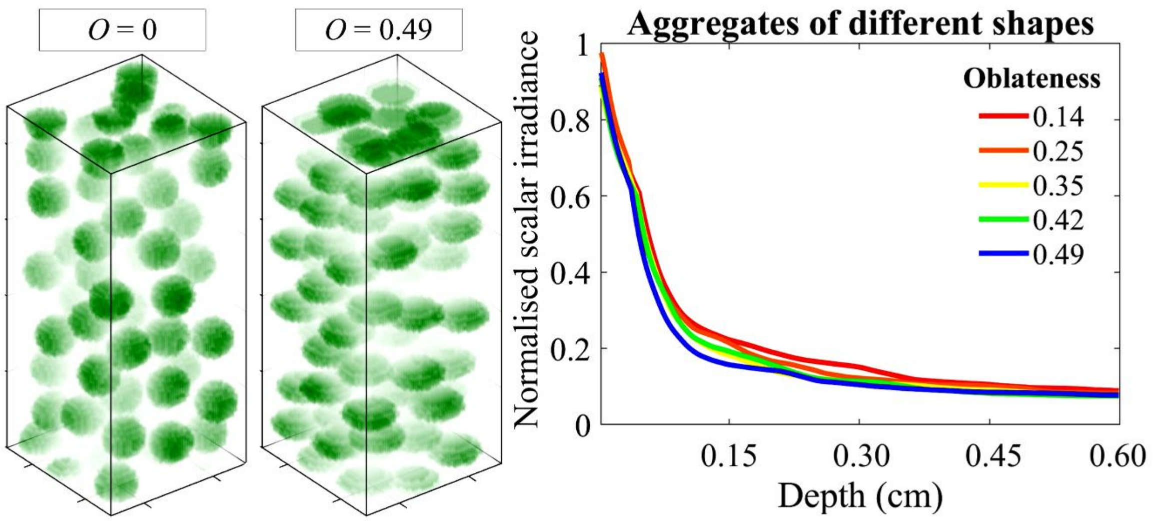

Figure S1. Simulation of the attenuation of scalar irradiance with depth in a hydrogel with microalgal aggregates of different oblateness ($O = \frac{a-c}{a}$), where *a* and *c* denote the major and minor axis, respectively.

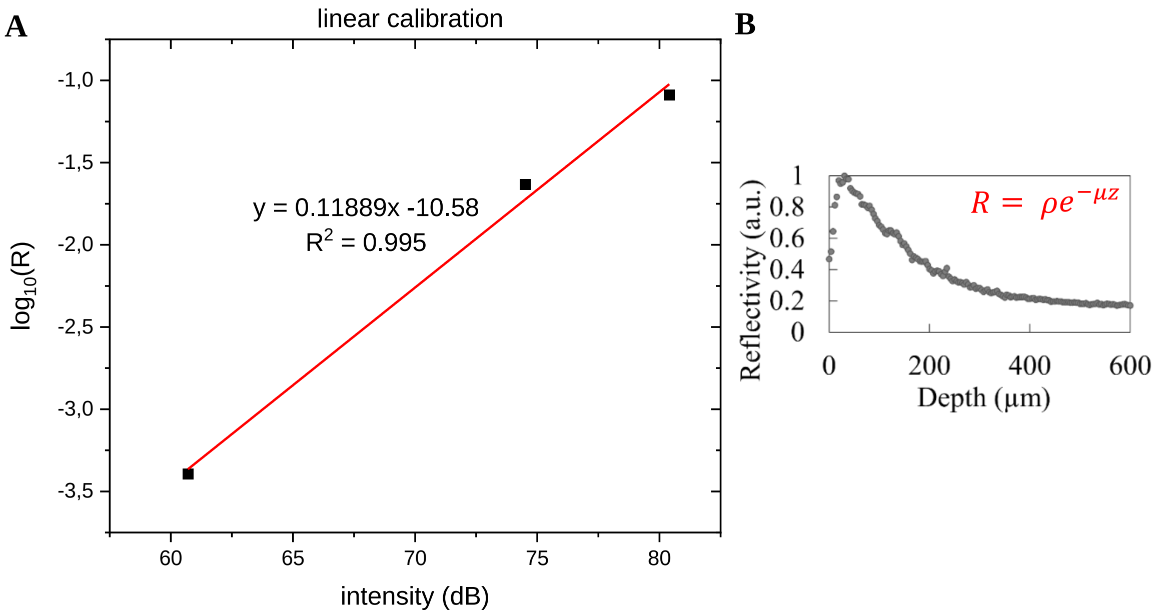

Figure S2. (A) Calibrated reflectivity versus OCT signal intensity in dB; (B) Reflectivity profile from an OCT scan of an algal aggregate illustrating the exponential decay of backscattered light with depth into the aggregate, which could be fitted with an empirical model (5).

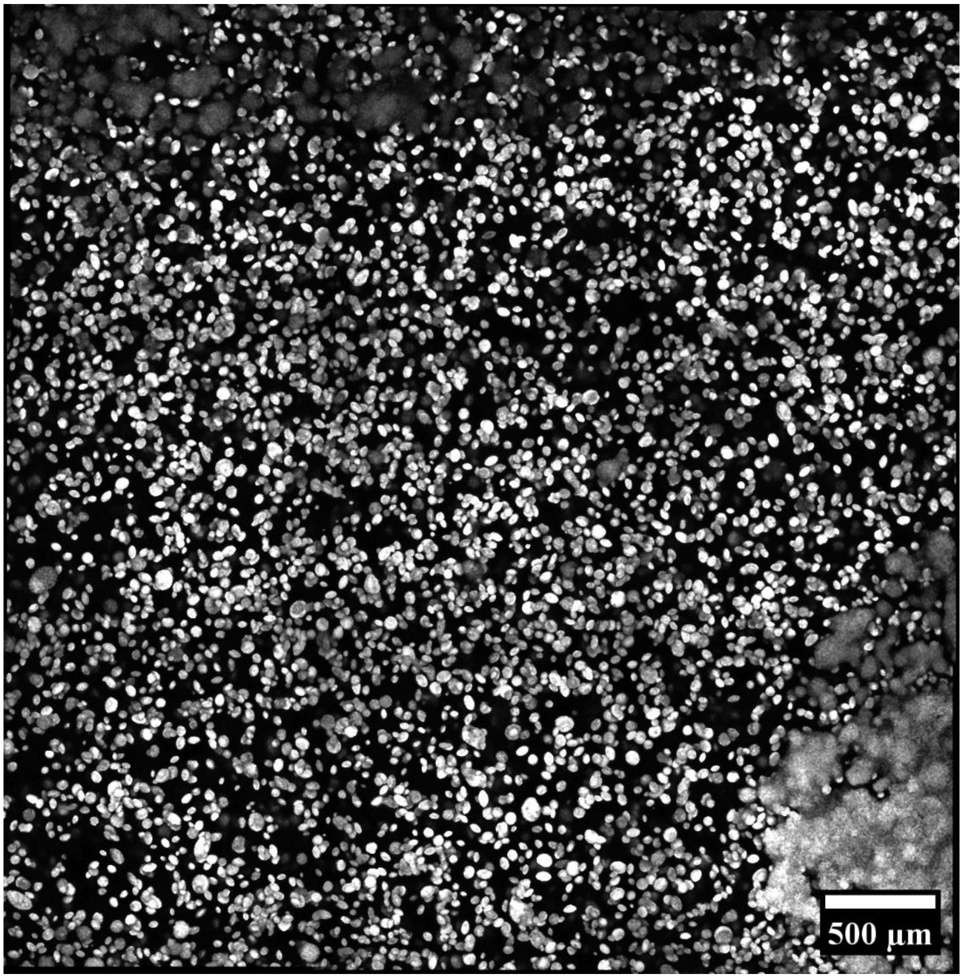

Figure S3. Confocal imaging (maximum intensity projection from a 100 µm deep Z stack) of *Chlorella vulgaris* aggregates after 7 days of growth within an agarose hydrogel.

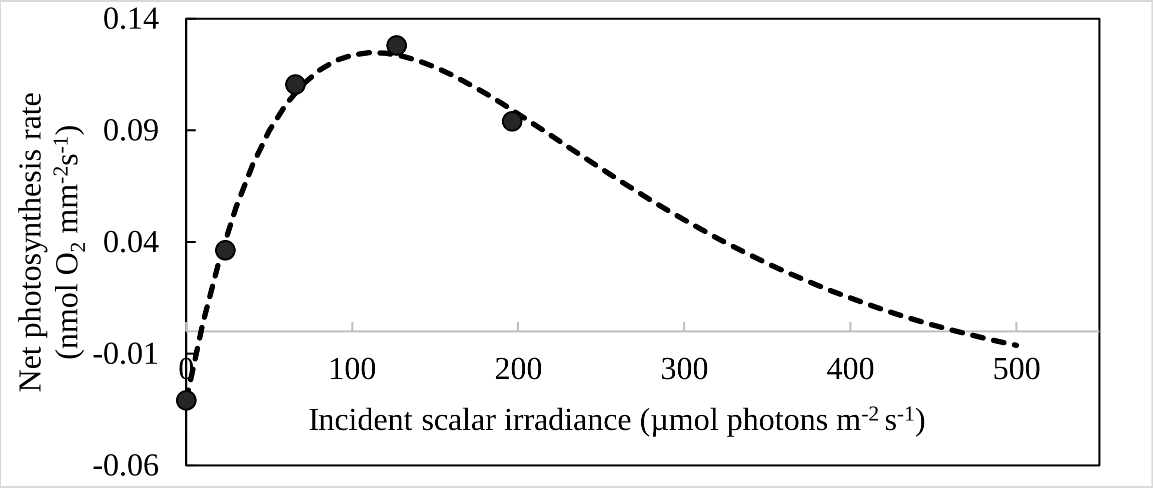

Figure S4. Light response curve obtained experimentally using microsensor measurements of photon scalar irradiance (400-700 nm) and oxygen concentration across an isolated *Chlamydomonas reinhardtii* aggregate within Tris-minimal medium, fitted to the empirical model of Platt et al. (6).

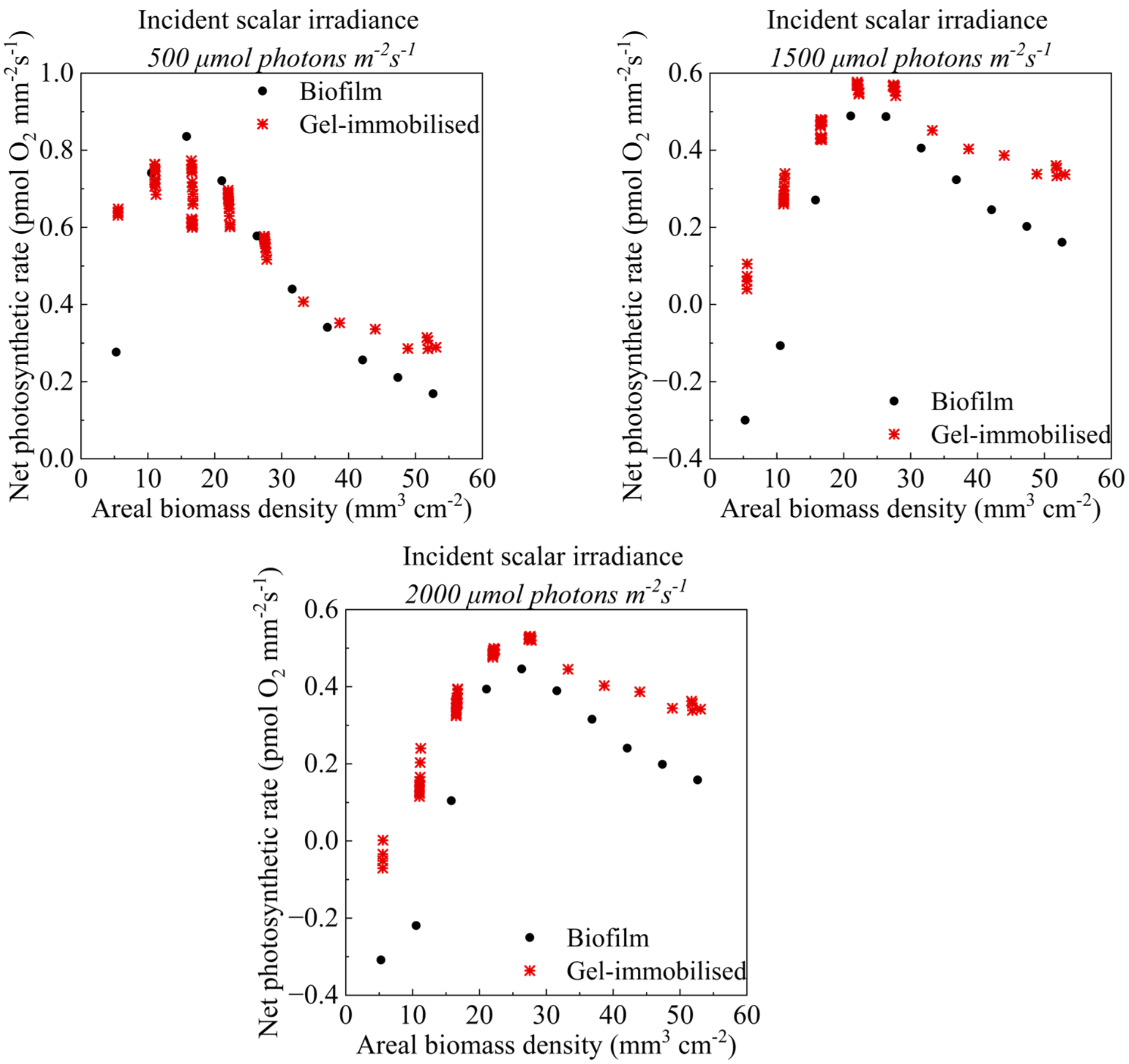

Figure S5. Calculation of the net photosynthetic rate by coupling the experimental light response curve to the simulated attenuation of photon scalar irradiance with depth among algal aggregates and through a biofilm of different areal biomass densities under an incident photon scalar irradiance (400-700 nm) of 500, 1500 and 2000 µmol photons m^{-2} s^{-1}, respectively.

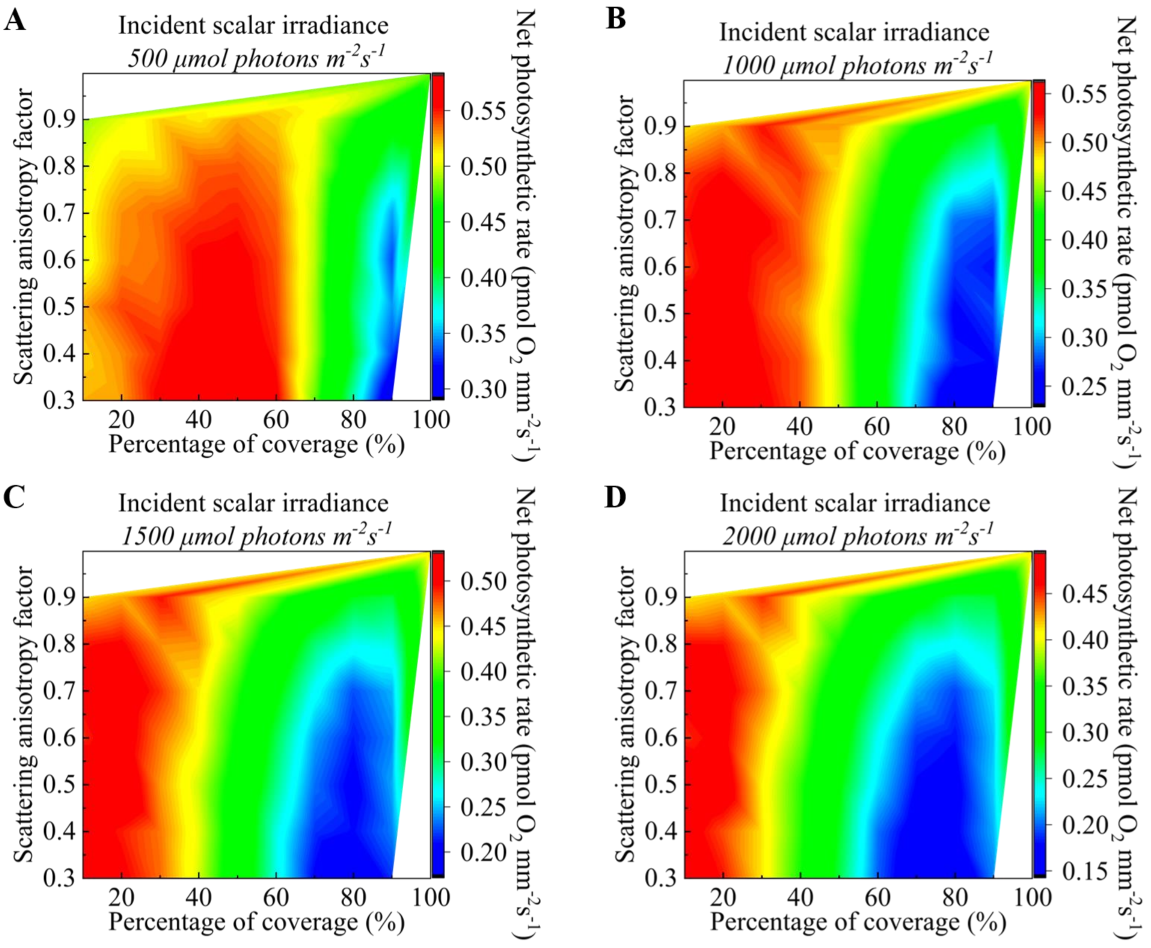

Figure S6. Calculation of the net photosynthetic rate by coupling the experimental light response curve to the simulated variation of normalised scalar irradiance among algal aggregates within different scattering matrix configurations under an incident photon scalar irradiance (400-700 nm) of (A) 500 µmol photons m^{-2} s^{-1}**;** (B) 1000 µmol photons m^{-2} s^{-1}**;** (C) 1500 µmol photons m^{-2} s^{-1}**;** (D) 2000 µmol photons m^{-2} s^{-1}.

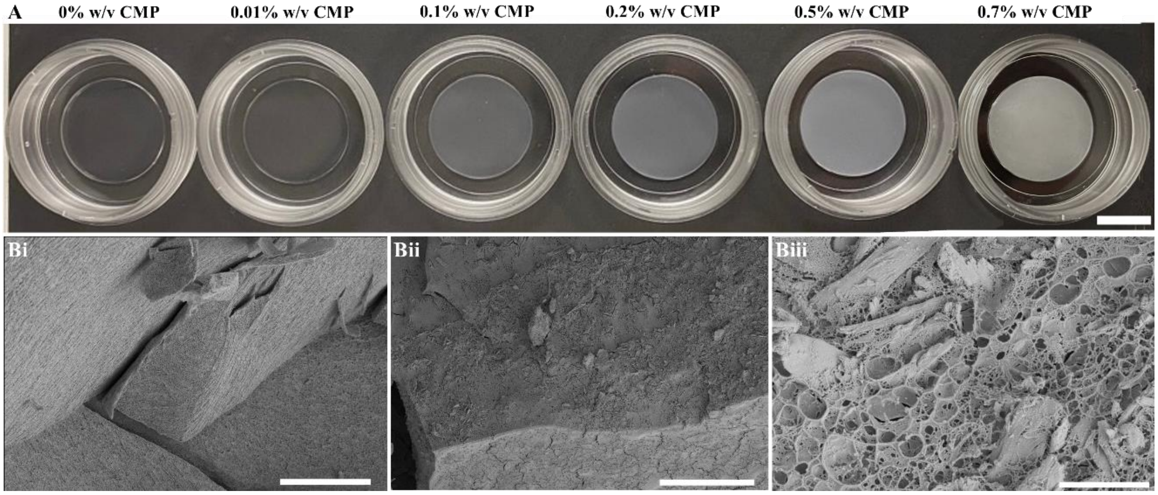

Figure S7. (A) Visual effect of cellulose microparticles (CMP) doping on agarose gel pads, with the weight percentage of CMP doping shown above the image. Scale bar = 1 mm. (B) Cryogenic scanning electron microscope images of hydrogels: (i) 0%w/v CMP (Scale bar = 20 µm); (ii) 0.7%w/v CMP (Scale bar = 20 µm) and (iii) 0.7%w/v CMP (Scale bar = 5 µm). The CMP scattering centers appear sheet-like among the agarose networks.

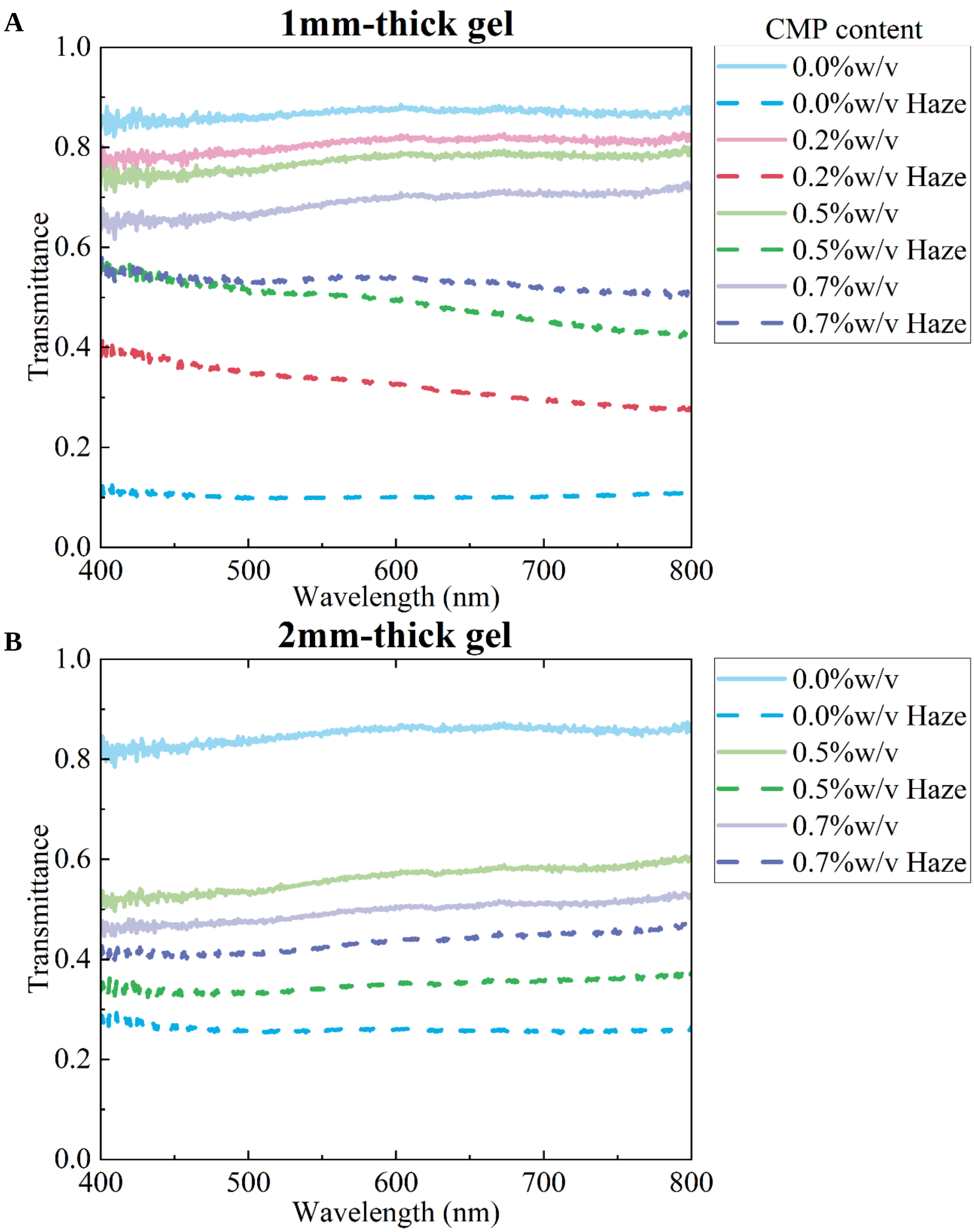

Figure S8. The effect of cellulose microparticles (CMP) doping on the transmission and scattering within 1 mm (A) and 2 mm (B) thick agarose hydrogels, across different levels of embedded CMP. Transmission spectra are shown with solid lines, haze spectra of scattered light are shown with dashed lines.

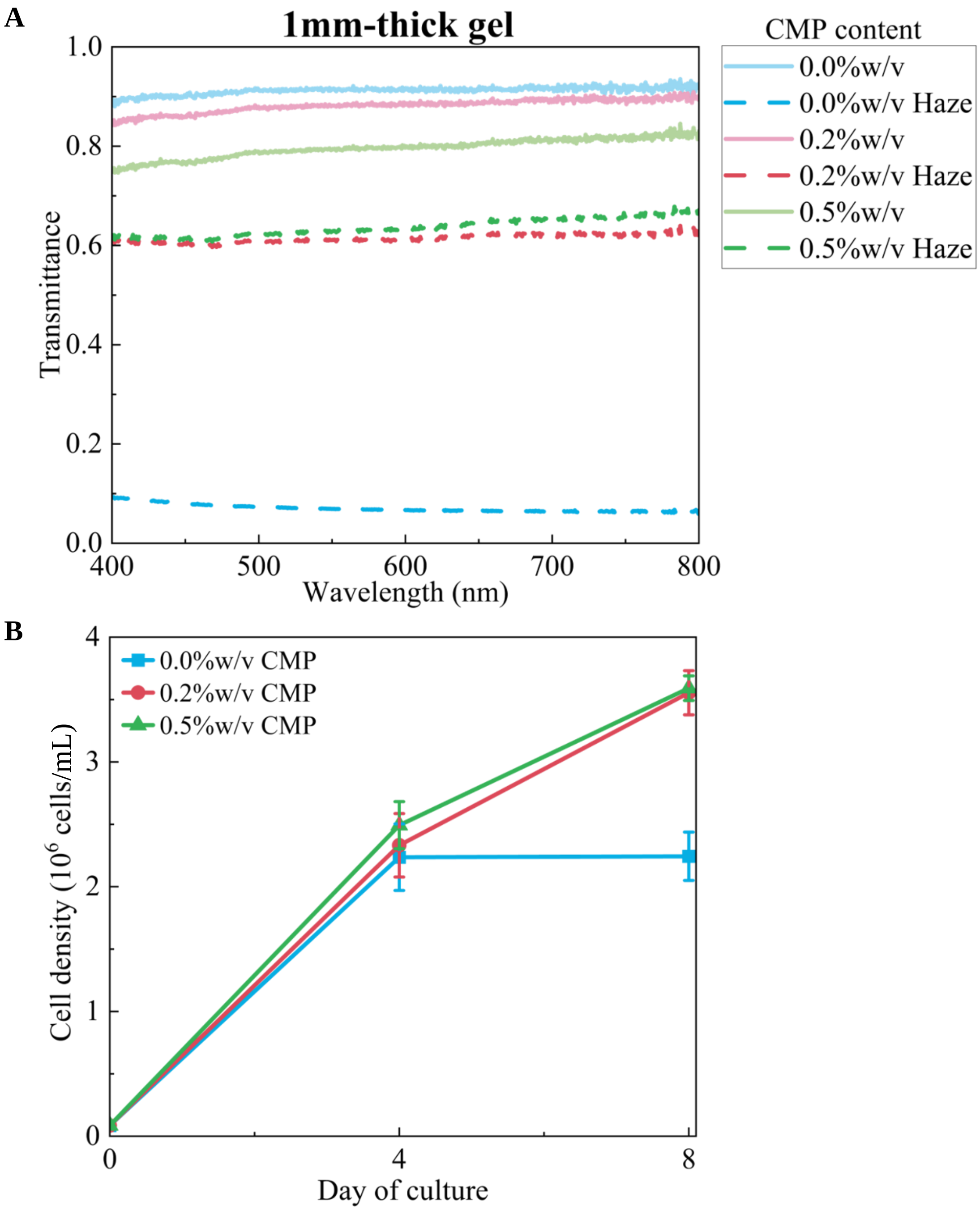

Figure S9. (A) The effect of cellulose microparticles (CMP) doping on the transmission and scattering within 1 mm-thick agarose hydrogels, across different levels of embedded CMP. Transmission spectra are shown with solid lines, spectra of scattered light are shown with dashed lines. (B) The cell growth curve of *Chlamydomonas reinhardtii* in 1 mm-thick gel pads of 0.5%wt agarose with Tris-minimal medium, colour matched to the CMP doping level it represents. It is noted how growth enhancement from CMP scattering was effective from day 4 onwards, as the culture was not yet light-limited during the initial growth phase with low cell density.

	Tris-minimal	Tris-minimal 0.5%w/v CMP	TAP	TAP 0.5%w/v CMP
Day 0	1.78 ± 0.05	1.78 ± 0.05	0.67 ± 0.04	0.67 ± 0.04
Day 4	3.0 ± 0.1	3.1 ± 0.2	2.07 ± 0.09	1.94 ± 0.06

Table S1. Cell density of *Chlamydomonas reinhardtii* in Tris-minimal and in TAP media with and without cellulose microparticles (CMP), before and after 4 days of incubation in the dark. The values correspond to the mean of nine replicates, in the unit of 10^6 cells per mL, with their respective standard error.

References

1. D. Levitz *et al.*, Determination of optical scattering properties of highly-scattering media in optical coherence tomography images. *Opt. Express* **12**, 249-259 (2004).
2. A. F. Fercher, W. Drexler, C. K. Hitzenberger, T. Lasser, Optical coherence tomography - principles and applications. *Reports on Progress in Physics* **66**, 239 (2003).
3. D. Wangpraseurt *et al.*, Microscale light management and inherent optical properties of intact corals studied with optical coherence tomography. *Journal of The Royal Society Interface* **16**, 20180567 (2019).
4. D. Levitz *et al.*, Quantitative characterization of developing collagen gels using optical coherence tomography. *Journal of Biomedical Optics* **15**, 026019 (2010).
5. L. J. Steven, S. Ravikant, C. Niloy, F. Yongji, L. David (2008) Measuring tissue optical properties in vivo using reflectance-mode confocal microscopy and OCT. in *Proc.SPIE*, p 68640B.
6. T. Platt, C. L. Gallegos, W. G. Harrison, Photoinhibition of photosynthesis in natural assemblages of marine phytoplankton. *Journal of Marine Research* **38**, 687-701 (1980).
